# Comparing the Clinical Utility of Rapid Diagnostics for Treatment of Bloodstream Infections Using Desirability of Outcome Ranking Approach for the Management of Antibiotic Therapy (DOOR-MAT)

**DOI:** 10.1128/AAC.00441-21

**Published:** 2021-08-17

**Authors:** Kimberly C. Claeys, Teri L. Hopkins, Kathryn Schlaffer, Stephanie Hitchcock, Yunyun Jiang, Scott Evans, J. Kristie Johnson, Surbhi Leekha

**Affiliations:** a Department Pharmacy Practice and Science, University to Maryland School of Pharmacy, Baltimore, Maryland, USA; b Department of Pharmacy, South Texas Veterans Health Care System, San Antonio, Texas, USA; c Department of Medicine, University of Maryland Medical Center, Baltimore, Maryland, USA; d Department of Pathology, University of Maryland School of Medicine, Baltimore, Maryland, USA; e Biostatistics Center and the Department of Biostatistics and Bioinformatics, The George Washington University, Washington, DC, USA; f Department of Epidemiology & Public Health, University of Maryland School of Medicine, Baltimore, Maryland, USA

**Keywords:** antibiotic stewardship, bloodstream infections, rapid diagnostic testing

## Abstract

Decisions regarding which rapid diagnostic test (RDT) for bloodstream infections to implement remain challenging given the diversity of organisms detected by different platforms. We used the desirability of outcome ranking management of antimicrobial therapy (DOOR-MAT) as a framework to compare two RDT platforms on potential desirability of antimicrobial therapy decisions. An observational study was performed at University of Maryland Medical System comparing Verigene blood culture (BC) to GenMark Dx ePlex blood culture ID (BCID) (research use only) panels on blood cultures from adult patients. Positive percent agreement (PPA) between each RDT platform and Vitek MS was calculated for comparison of on-panel targets. Theoretical antimicrobial decisions were made based on RDT results, taking into consideration patient parameters, antimicrobial stewardship practices, and local infectious diseases epidemiology. DOOR-MAT with a partial credit scoring system was applied to these decisions, and mean scores were compared across platforms using a paired *t* test. The study consisted of 160 unique patients. The Verigene BC PPA was 98.6% (95% confidence interval [CI], 95.1 to 99.8), and ePlex BCID PPA was 98% (95% CI, 94.3 to 99.6). Among the 31 organisms not on the Verigene BC panels, 61% were identified by the ePlex BCID panels. The mean (standard deviation [SD]) DOOR-MAT score for Verigene BC was 86.8 (28.5), while that for ePlex BCID was 91.9 (23.1) (*P = *0.01). Both RDT platforms had high PPA for on-panel targets. The ePlex BCID was able to identify more organisms than Verigene, resulting in higher mean DOOR-MAT scores.

## INTRODUCTION

Molecular diagnostic tests for the management of bloodstream infections (BSIs) in the clinical microbiology laboratory are rapidly evolving (1). In particular, the use of rapid diagnostic tests (RDTs) in BSI has been shown to optimize antibiotic therapy selection (2–5), preventing both under-treatment with ineffective agents, which could lead to poor patient clinical outcomes, and overtreatment with agents that are overly broad in spectrum, which could lead to selective pressure and development of antibiotic resistance. There are multiple molecular RDTs that can identify organisms and key genetic resistance mechanisms hours and even days sooner than traditional methods (2–4, 6, 7). Our ability as health care providers, however, to understand and optimally implement these platforms has not kept pace with the available RDT technology (8, 9).

Comparisons between RDT platforms are largely limited to *in vitro* studies of sensitivity and specificity of on-panel organisms (10–12). These provide little insight regarding how RDT results could be interpreted and applied to affect antimicrobial therapy selection and downstream clinical outcomes. The ability to make these pragmatic comparisons, however, has become increasingly important given the availability of multiple commercial RDT platforms and difficulty in comparing platforms based on considerations relevant to an individual’s institution.

The Antibiotic Resistance Leadership Group (ARLG) developed the method called “desirability of outcome ranking management of antimicrobial therapy” (DOOR-MAT), which provides a framework to compare desirability of potential antimicrobial therapy decisions as a function of final organism identification and phenotypic susceptibility profile (13). Use of DOOR-MAT allows ranking of antimicrobial therapy decisions based on antimicrobial stewardship (AMS) goals to improve clinical outcomes while reducing selective pressure and antimicrobial resistance (14). This ranking is used in conjunction with partial credit scoring to allow for quantitative comparisons of the clinical desirability of RDT-based decisions. Through the use of DOOR-MAT, institutions can compare RDTs prior to implementation based on local infectious disease epidemiology, antimicrobial prescribing patterns, and AMS principles. Few studies to date, however, have incorporated this novel methodology, and only one has focused on RDTs commonly used in clinical practice (15). The objective of the current study was to compare the theoretical antimicrobial treatment decisions guided by two commercially available RDT platforms used in the management of BSI and evaluate the potential clinical utility through using the DOOR-MAT approach.

(This work was presented in part as a poster presentation at ID Week, Washington, DC, October 2019.)

## RESULTS

### RDT discrepancy analysis and panel performance.

The study consisted of 174 positive blood cultures from unique patients that were tested on both RDT platforms. Fourteen samples were excluded for the following reasons: pediatric patient (*n* = 1), Verigene BC panel not performed for comparison (*n* = 6), outpatient (*n* = 3), invalid information at collection (*n* = 2), not enough sample available for discrepancy analysis (*n* = 2). This left 160 samples for further analysis; 90 on Gram-negative panels and 70 on Gram-positive panels. Discrepancy testing by GenMark Diagnostics was performed on 11 samples (Table S1); one sample was of insufficient quantity to complete further testing.

Of the 90 blood samples tested on Gram-negative panels, three had two Gram-negative rods identified, for a total of 93 Gram-negative organisms. Before discrepancy analysis, the GenMark Dx ePlex blood culture ID (Gram-negative) (BCID-GN) research use only (RUO) panel missed 1 Pseudomonas aeruginosa, 2 Stenotrophomonas maltophilia, and 1 Escherichia coli isolate for a positive percent agreement (PPA) of 95.2% (95% confidence interval [CI], 88.3 to 98.7). After discrepancy analysis, the ePlex BCID-GN panel misidentified one S. maltophilia isolate for a PPA of 98.9% (95% CI, 93.5 to 99.9). The only Gram-negative resistance determinant detected during this study was CTX-M (*n* = 6) and was identified by both panels correctly in 100% of samples. Of note, the pan-Gram-positive target was positive in 5 samples tested on the ePlex BCID-GN panel; of these, 3 were determined to be true positives. In comparison, the Verigene BC-GN did not identify 1 Klebsiella pneumoniae isolate, for a PPA of 98.6% (95% CI, 92.7 to 99.9).

Among the Gram-negative organisms identified by Vitek 2 MS, 9 (9.7%) were off-panel organisms that could not have been detected by the ePlex BCID-GN panel: Acinetobacter junii (*n* = 1), Achromobacter denitrificans (*n* = 1), Achromobacter xylosoxidans (*n* = 1), Burkholderia cepacia complex (*n* = 1), Pasteurella multocida (*n* = 2), Prevotella intermedia (*n* = 1), *Psychrobacter* spp. (*n* = 1), and Pseudomonas pseudoalcaligenes (*n* = 1). Among the Gram-negative organisms identified by Vitek 2 MS, 20 (21.5%) were off-panel organisms that could not have been detected by Verigene BC-GN: Achromobacter denitrificans (*n* = 1), Achromobacter xylosoxidans (*n* = 1), Bacteroides fragilis (*n* = 1), Burkholderia cepacia complex (*n* = 1), Morganella morganii (*n* = 1), Pasteurella multocida (*n* = 2), Prevotella intermedia (*n* = 1), Pseudomonas pseudoalcaligenes (*n* = 1), *Psychrobacter* spp. (*n* = 1), Serratia marcescens (*n* = 7), and *Stenotrophomonas* spp. (*n* = 3). Among the 20 Gram-negative organisms that were not on the panel for Verigene BC-GN, 55% were identified by the ePlex BCID-GN panel.

Of the 70 blood samples tested on Gram-positive bottles, 3 had two Gram-positive organisms, resulting in 73 total. Before discrepancy analysis, the ePlex BCID-GP RUO panel missed or misidentified 1 Staphylococcus epidermidis, 1 Staphylococcus aureus, and 1 Enterococcus faecalis, for a PPA of 97.2% (95% CI, 90.1 to 99.7). After discrepancy analysis was performed, the ePlex BCID-GP panel demonstrated a 98.6% PPA (95% CI, 92.4 to 99.9). Both *vanA* (*n* = 4) and *mecA* (*n* = 27) were detected correctly. Of note, the pan-Gram-negative probe was positive in 1 sample tested on the ePlex BCID-GP panel; however, after further testing on blood and isolated colonies, a Gram-negative organism was not detected, resulting in a false-positive. The Verigene BC-GP missed 1 Streptococcus mitis/Streptococcus oralis, for a PPA of 98.3% (95% CI, 91.1 to 99.9).

Among the Gram-positive organisms identified by Vitek 2 MS, 3 (4.1%) were off-panel organisms that could not have been detected by the ePlex BCID-GP panel: ID resembling *Lactobacillus* (*n* = 1), Globicatella sanguinis (*n* = 1), and Eggerthella lenta (*n* = 1). Among the Gram-positive organisms identified by Vitek 2 MS, 11 (14.8%) were off-panel organisms that could not have been detected by Verigene BC-GP: *Micrococcus* spp. (*n* = 6), Enterococcus gallinarum (*n* = 1), *Corynebacterium* spp. (*n* = 1), ID resembling *Lactobacillus* (*n* = 1), Globicatella sanguinis (*n* = 1), and Eggerthella lenta (*n* = 1). Among the 11 Gram-positive organisms that were not on the panel for Verigene BC-GP, 64% were identified by the ePlex BCID-GP panel.

The overall PPA for ePlex BCID panels was 98% (95% CI, 94.3 to 99.6), and that for Verigene BC panels was 98.6% (95% CI, 95.1 to 99.8). Among the 31 organisms that were not on the panel for Verigene BC, 61% were identified by ePlex BCID panels.

### DOOR-MAT analysis.

All 160 samples were tested on both RDT platforms and had sufficient clinical information for assessment with DOOR-MAT (Table 1). The median age of patients was 56 years (interquartile range [IQR], 41 to 66), and the majority were male (66.3%). The most common sources of BSI were genitourinary and unknown. The most common empirical antibiotics targeted toward the identified organisms were vancomycin (31.2%) and piperacillin-tazobactam (45.4%).

**TABLE 1:** Cohort demographics and baseline clinical data

Characteristic^*a*^	No. (%) (*n* = 160)
Allergy history	
PCN	17 (10.6)
Cephalosporin	3 (1.9)
Sulfa antibiotic	6 (3.8)
Other antibiotic^*b*^	8 (5)
Prior MDRO	
CRE	2 (1.3)
ESBL	5 (3.2)
MDR-PSA	1 (0.6)
MRSA	23 (14.4)
VRE	6 (3.8)
Intensive care unit at BSI	111 (69.4)
ID consult with 24 h of blood culture positivity	61 (38.1)
Polymicrobial BSI	10 (6.3)
Source of BSI	
Bone/joint	2 (1.3)
Cardiac	3 (1.9)
Contaminant^*c*^	27 (16.9)
Endovascular	14 (8.8)
Genitourinary	34 (21.3)
Intra-abdominal	17 (10.6)
Other	3 (1.9)
Respiratory	9 (5.6)
Skin/soft tissue	12 (7.5)
Unknown	39 (24.4)
Hospital service	
Cardiology	15 (9.4)
Medicine/hospitalist	61 (38.1)
Oncology	21 (13.1)
Shock/trauma	20 (12.5)
Surgical	12 (7.5)
Transplant	21 (13.1)
Other	10 (6.3)

a PCN, penicillin; MDRO, multidrug-resistant organism; CRE, carbapenem-resistant *Enterobacterales*; ESBL, extended-spectrum beta-lactamase; MDR-PSA, multidrug-resistant P. aeruginosa; MRSA, methicillin-resistant S. aureus; VRE, vancomycin-resistant enterococcus; BSI, bloodstream infection; ID, infectious disease.

b Other antibiotics included doxycycline, fluoroquinolones, and daptomycin.

c Contaminant and not a cause of infection, as noted in the patient’s medical chart.

**FIG 1:**
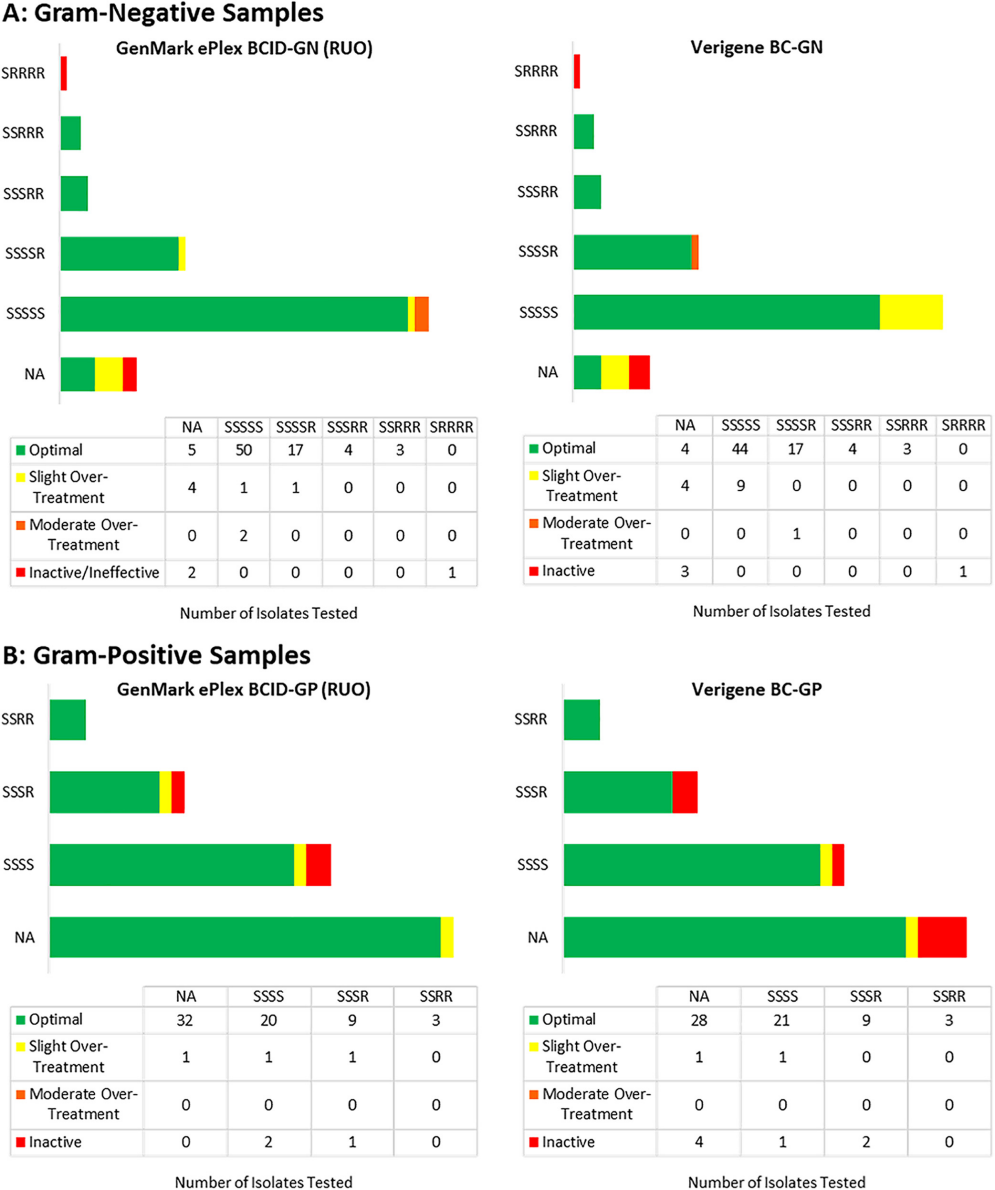
Clinical isolates by final susceptibility phenotype profile among Gram-negative and Gram-positive organisms by treatment based on results of ePlex BCID panels versus Verigene BC. Comparison of theoretical antibiotic management decisions based on GenMark ePlex BCID RUO or Vergine BC results by final organism susceptibility profile. NA, no routine susceptibility testing (e.g., coagulase-negative *Staphylococcus*) or uncommon profile (e.g., *Stenotrophomonas maltophilia*).

Overall agreement between ID clinicians on assignment for the most desirable antimicrobial treatment based on Verigene BC results was 81% (95% CI, 70 to 92). Overall agreement on assignment for most desirable antimicrobial treatment based on the ePlex BCID panel results was 71% (95% CI, 53 to 91). Antimicrobial therapy decisions were optimal for the majority of both Gram-positive and Gram-negative organisms based on decisions made by with both RDT platforms (Fig. 1).

The overall mean score for Verigene BC panels was 86.8 (standard deviation [SD], 28.5), while the overall mean score for ePlex BCID panels was 91.9 (SD, 23.1) (*P = *0.01). Among Gram-negative organisms, the mean (SD) score for Verigene BC-GN panel was 85.4 (SD, 26.6), while the mean score for ePlex BCID-GN panel was 90.7 (SD, 23.7) (*P = *0.03). Among Gram-positive organisms, the mean score for the Verigene BC panel was 88.6 (SD, 30.9), while the overall mean score for the ePlex BCID-GP panel was 93.6 (SD, 22.4) (*P = *0.13).

## DISCUSSION

In this observational study of 160 clinical blood cultures from adult patients, both the Verigene BC and the ePlex BCID RUO panels performed well with respect to on-panel organism identification. Of interest, however, was that the ePlex BCID panels were able to identify most organisms in the study sample and more than Verigene BC. This is due to fewer organisms being on current Verigene GP or GN panels. Our findings suggest that detection of more organisms by GenMark ePlex BCID translates into potentially improved antimicrobial therapy decisions, as reflected in higher DOOR-MAT scores, particularly among Gram-negative BSIs.

The PPA demonstrated in this research, both overall and individually for Gram-positive and Gram-negative organisms, aligned with previously published research examining platform agreement with reference standards (16, 17). In a recent multicenter laboratory study of the ePlex BCID panels, 2,342 Gram-positive samples were tested (17). Investigators reported an overall on-panel agreement of 89% before discordant sample resolution and the final overall weighted PPA across targets of 96%. Recently, the ePlex BCID RUO panels were compared to the BioFire FilmArray blood culture ID (BCID) panel in 137 clinical blood cultures (18). Among the 98 Gram-positive and 33 Gram-negative organisms included, agreement on final organism identification was over 98% for both RDT platforms.

An important difference between these panels is the presence of the pan-target probes on the ePlex BCID panels, which help to identify organisms that may be Gram variable or cause polymicrobial infections that may be missed when selecting a panel after Gram stain analysis. Among the samples tested in this study, few were pan-target positive, and half were determined to be false positives after additional discrepancy testing. In a previous laboratory study of the ePlex BCID-GP panel assessing the clinical study performed for regulatory clearance of the ePlex BCID-GP panel, the PPA of the pan-Gram-negative target was 95.7%, which does not align with current findings from testing the ePlex RUO BCID-GP panels, but our sample size was limited.

Currently, comparisons between RDT platforms are largely limited to *in vitro* analysis of on-panel organisms (9–11), which does not provide insight into how each RDT platform would change clinical decision making. The DOOR-MAT provides a framework for institutions to compare RDTs and their potential impact on antimicrobial therapy decisions without the need to fully implement both platforms. The desirability of antimicrobial decisions in DOOR-MAT, though broadly based on *in vitro* activity, can be adapted to incorporate both institutional AMS guidelines and local infectious diseases epidemiology. In this study, the GenMark ePlex BCID had a higher mean DOOR-MAT score due to the expanded number of targets identified. While this could be inferred by *in vitro* analysis, the added benefit of DOOR-MAT is in knowing the extent to which these panel differences could impact decision-making in the context of which organisms impact your local patient population.

The partial credit scoring function also allows assessment of antibiotic desirability through comparison of multiple approaches wherein scores for decisions can be adapted based on institutional priorities and balances of risks of adverse events or resistance versus benefits on clinical outcomes such as inpatient mortality or length of inpatient stay. For example, treatment of potential AmpC organisms with cefepime versus piperacillin-tazobactam or treatment of *Micrococcus* with an antibiotic versus treated as a contaminant can be scored differently in different scoring models. Additionally, multivariable modeling of DOOR-MAT scores as a function of resistance patterns, institutional guideline adherence, and relative clinical importance can be considered in future analyses (19). As such, DOOR-MAT has the potential to be a powerful AMS tool.

This study is not without limitations. Since this is a theoretical analysis, the results cannot be directly extrapolated to patient clinical outcomes. Although the GenMark ePlex BCID demonstrated statistically higher mean DOOR-MAT scores than Verigene BC, both the immediate impact on an individual patient’s clinical outcome and the long-term impact on antimicrobial resistance is unknown. This theoretical comparison also does not incorporate real-world considerations such as algorithm adherence or use of nonrecommended agents, e.g., fluoroquinolones. Review of clinical scenarios and RDT results in this study was completed by ID-trained clinicians; however, many institutions rely on front-line providers for interpretation and action, which could produce different results (8). Generalizability of results may be further limited by the influence of local epidemiology of BSIs. Additionally, the small sample size limits the ability for a more granular assessment of specific clinical scenarios that may be of interest. For example, Verigene BC is known to underperform in polymicrobial BSIs, but those comparisons are difficult to perform with the small number of polymicrobial BSIs in this sample (20).

The strength of DOOR-MAT is that it is a low-resource method to compare testing platforms accounting for institutional infectious disease epidemiology and AMS principles. Ideally, some form of implementation would need to be completed to have a more thorough comparison of RDTs and truly assess clinical outcomes. However, this is resource intensive and often not practical, and in lieu of a randomized or quasiexperimental evaluation comparing clinical outcomes, this theoretical use of DOOR-MAT allows a pragmatic glimpse into the potential differences between platforms (21).

In summary, the current study provides a functional example of using the DOOR-MAT framework with partial credit scoring to provide a quantitative comparison of the potential impact of two commercially available RDT platforms among clinical blood culture samples.

## MATERIALS AND METHODS

### Population and study setting.

This was an observational study at two acute care hospitals within University of Maryland Medical System (UMMS). Patients were adults (age ≥ 18 years) with at least one positive blood culture with a bacterial organism(s) detected on Gram staining. The study consisted of consecutive positive blood cultures obtained from March 2018 to July 2019. To allow an appropriately large sample of Gram-negative organisms, cultures were included in a fixed 1:1 ratio of Gram-positive to Gram-negative organisms. Patients were excluded if Gram staining demonstrated yeasts or no organism, if blood cultures were collected from an outpatient facility, or if a discarded sample was not available for additional molecular testing. Only the first occurrence of BSI during the same hospitalization was collected and included in the study. The study was approved by the University of Maryland Institutional Review Board with a waiver of informed consent.

### Standard microbiological processing.

During the study period, blood culture bottles for the two acute care hospitals were sent to the UMMS Central Microbiology Laboratory for organism identification and susceptibility testing. Growth of organisms in blood culture bottles was first detected by BacTAlert automated 3D system (BacT/Alert aerobic SA and anaerobic SN bottles, bioMérieux Durham, NC). Immediately following detection of organisms, Gram stain microscopy was performed. If Gram stain resulted in identification of Gram-positive and/or Gram-negative bacteria, two aliquots of the sample were transferred to the appropriate Verigene blood culture (BC) panel, BC-GN or BC-GP (Luminex Corporation, Austin, TX, USA). Bacterial identification from the bacterial colonies was performed using Vitek MS and susceptibility testing using the Vitek 2 automated susceptibility testing (AST) system (bioMérieux, Durham, NC), which served as the final reference method. All results of Gram staining, Verigene BC results, and final susceptibilities were part of routine clinical care and reported to the provider and documented in the electronic medical record (EMR).

### Experimental RDT testing.

The GenMark Dx ePlex blood culture identification (BCID) (research use only) Gram-positive (BCID-GP) and Gram-negative (BCID-GN) panels were used for retained blood samples that were frozen at −80°C. Samples were thawed in batches of 12 and run using the GenMark Dx ePlex system (GenMark Diagnostics, Inc., Carlsbad, CA, USA). The ePlex BCID panels are multiplex nucleic acid amplification assays that detect 20 Gram-positive organisms at the genus or species level, including four resistance genes, and 21 Gram-negative organisms at the genus or species level, including six resistance genes (16). A comparison of organisms detected by the Verigene BC and GenMark Dx ePlex BCID panels is detailed in Table 2. Results for the ePlex BCID panels were documented in a password-protected database and not made available to patients’ medical providers. While the ePlex RUO BCID panels were utilized for this study, the BCID panels have since achieved 510k clearance from the FDA. There are no differences between the two products, with the exception of the Klebsiella pneumoniae reported result being expanded to the K. pneumoniae group, which now includes Klebsiella variicola and Klebsiella quasipneumoniae.

**TABLE 2:** Comparison of RDT platforms

Target type	Verigene blood culture	GenMark Dx ePlex blood culture ID	
		Similar to Verigene BC	ePlex BCID only
Gram-positive bacteria	Enterococcus faecalis, Enterococcus faecium, Staphylococcus aureus, Staphylococcus epidermidis, Staphylococcus lugdunensis, Streptococcus agalactiae, Streptococcus anginosus, Streptococcus pneumoniae, Streptococcus pyogenes, *Listeria* spp., Staphylococcus spp., Streptococcus spp.	Enterococcus faecalis, Enterococcus faecium, Staphylococcus aureus, Staphylococcus epidermidis, Staphylococcus lugdunensis, Streptococcus agalactiae, Streptococcus anginosus, Streptococcus pneumoniae, Streptococcus pyogenes, *Listeria* spp., Staphylococcus spp., Streptococcus spp.	Bacillus cereus, Bacillus subtilis, Cutibacterium acnes, Listeria monocytogenes, *Corynebacterium* spp., *Enterococcus* spp., *Lactobacillus* spp., *Micrococcus* spp.
Gram-negative bacteria	Pseudomonas aeruginosa, Escherichia coli, Klebsiella pneumoniae, Klebsiella oxytoca, Acinetobacter spp., *Citrobacter* spp., Enterobacter spp., Proteus spp.	Escherichia coli, Klebsiella oxytoca, Klebsiella pneumoniae, Pseudomonas aeruginosa, *Citrobacter* spp., Enterobacter spp., Proteus spp.	Acinetobacter baumannii, Bacteroides fragilis, Cronobacter sakazakii, Enterobacter cloacae, Fusobacterium nucleatum, Fusobacterium necrophorum, Haemophilus influenzae, Morganella morganii, Neisseria meningitidis, Proteus mirabilis, Serratia marcescens, Stenotrophomonas maltophilia, Salmonella spp., *Serratia* spp.
Resistance determinants	*mecA, vanA/B*; CTX-M, KPC, IMP, VIM, OXA, NDM	*mecA, mecC, vanA, vanB*; CTX-M, KPC, IMP, VIM, OXA, NDM	
Other	NA	Pan-Gram-negative probe (BCID-GP panel only), pan-Gram-positive probe (BCID-GN panel only); pan-*Candida* probe (both panels)	

### Resolution of discrepancies.

The results of the ePlex BCID panels were compared to those obtained from final organism identification and phenotypic susceptibilities using Vitek MS and Vitek 2 AST. Discrepancies were defined as organism or resistance determinants which were targets on the panels of interest that were detected on final culture but not on the RDT panel (false negatives) or detection of organisms or resistance determinants on the RDT panel that were not identified in the final culture (false positives). Frozen 1-ml aliquots were used for discrepancy testing, which included in-house repeat panel testing. Microbiological discrepancies were further investigated by GenMark Diagnostics (Carlsbad, CA). Twelve frozen blood samples were sent to GenMark Diagnostics. The samples underwent additional analysis, including subculturing on selective and nonselective agar and molecular tests such as PCR, 16S sequencing, and amplification sequencing.

### Data collection.

Patient baseline demographic data, comorbid conditions, source of BSI, antimicrobial allergies, and history of multidrug-resistant infection/colonization were obtained from review of the EMR. Additionally, data on final organism identification and phenotypic susceptibility data from Vitek MS and Vitek 2 AST were also collected from the EMR.

### Development of DOOR-MAT framework and scoring.

**FIG 2:**
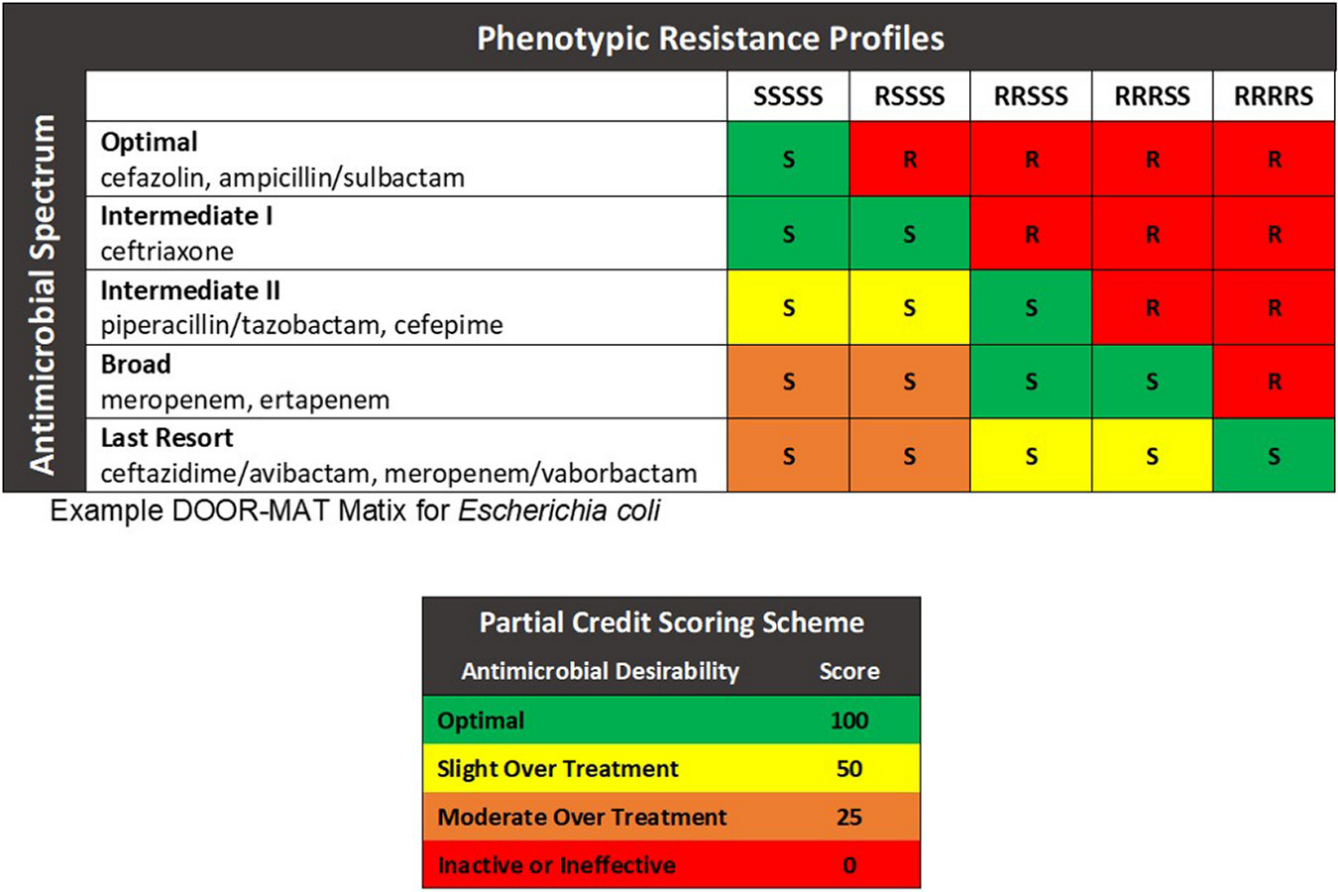
Example of the DOOR-MAT framework and partial credit scoring system.

To develop DOOR-MAT frameworks, we first determined antimicrobials commonly used at our institution and ranked them on their known spectra of activity (19). For instance, for treatment of BSI caused by E. coli, common beta-lactams that may be considered for therapy, ranked from most narrow to most broad, included cefazolin or ampicillin/sulbactam, ceftriaxone, piperacillin-tazobactam or cefepime, meropenem or ertapenem, and finally ceftazidime-avibactam or meropenem/vaborbactam. Then, for each organism or group of organisms under study, we considered common phenotypic resistance profiles that would result from AST. For example, resistance profiles could range from susceptible to all considered antimicrobials (S-S-S-S-S) to susceptible only to agents of last resort (R-R-R-R-S). By cross-referencing the antimicrobial spectra of activity and organism phenotypic resistance profiles, we then created DOOR-MAT matrices (Fig. 2). Using these matrices, antimicrobial therapy decisions were then defined as optimal, various degrees of suboptimal (e.g., slight overtreatment), or inactive/ineffective and a partial credit scoring system was then applied. The partial credit scoring used in this study assigned zero points to inactive/ineffective therapy and 100 to optimal, most narrow, therapy.

**FIG 3:**
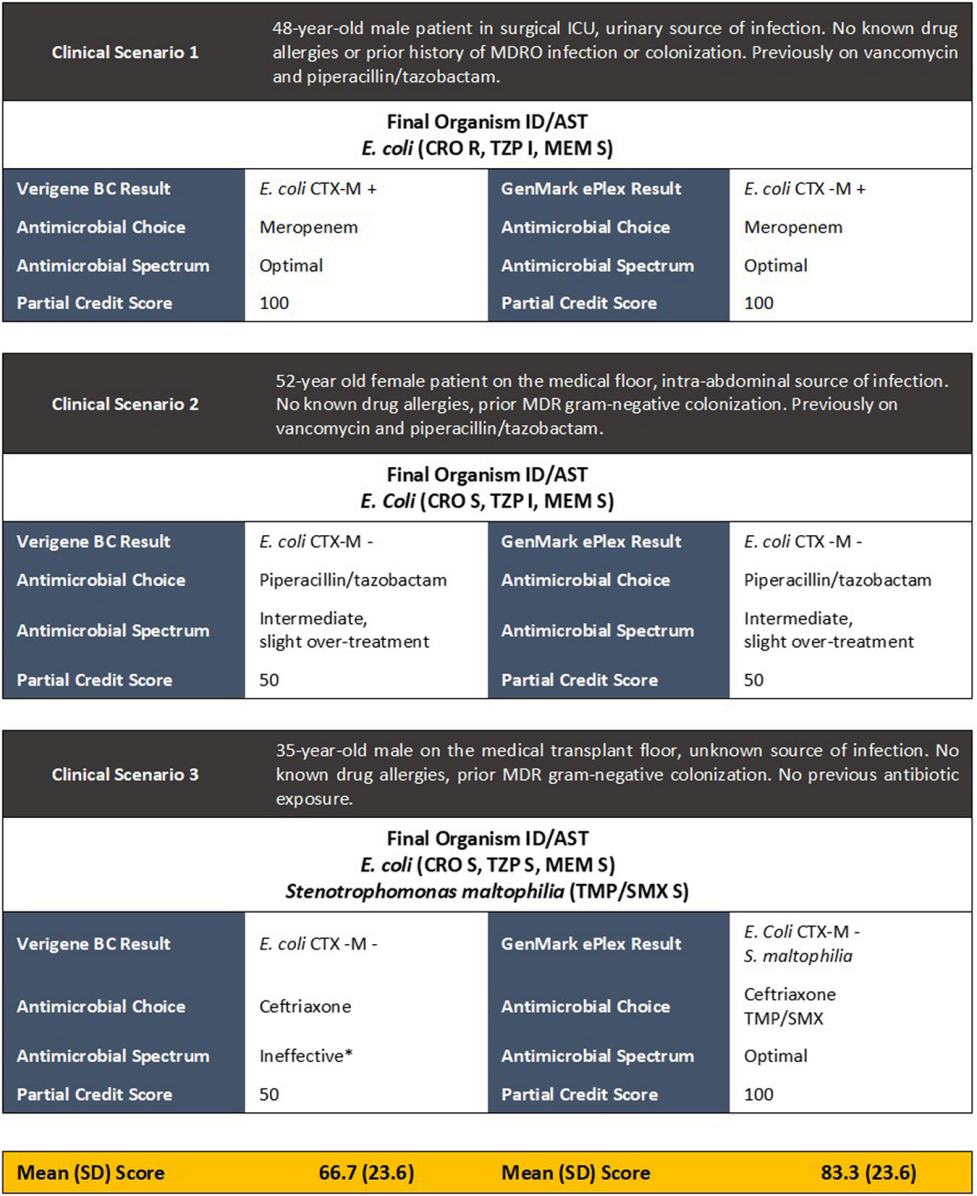
Example of clinical scenarios and application of DOOR-MAT and partial credit scoring. *, not effective for the treatment of one or more organisms in final culture. AST, antimicrobial susceptibility testing; BC, blood culture; CRO, ceftriaxone; ICU, intensive care unit; MDRO, multidrug resistant organism; MEM, meropenem; TZP, piperacillin-tazobactam.

To provide a functional example, the DOOR-MAT matrices presented in Fig. 2, which was developed based on local institutional infectious diseases epidemiology and prescribing practices, can then be applied to a series of RDT results for blood cultures identifying a variety of organisms. The results of each respective RDT could lead to different prescribing decisions based on available information, such as organism and/or resistance determinants detected. In real-world situations, these decisions would be supplemented with patient-specific information, such as previous antibiotic exposure. Figure 3 provides examples of application of the DOOR-MAT matrix for optimal therapy of E. coli based on final susceptibility profile and the corresponding partial credit score. Partial credit scoring allows for introduction of context for nuanced clinical and AMS considerations, the full discussion of which is beyond the scope of the current paper (19).

These DOOR-MAT matrices were made individually *a priori* for Staphylococcus spp., *Enterococcus* spp., Acinetobacter spp., Pseudomonas aeruginosa, and on-panel *Enterobacterales* (E. coli, Klebsiella spp., *Citrobacter* spp., and Enterobacter spp.). For all other on-panel organisms (i.e., Stenotrophomonas maltophilia and *Micrococcus* spp.), scoring was based on *in vitro* susceptibility, wherein a score of 100 was assigned if the organism was *in vitro* susceptible and a score of 0 was used for inactive or unnecessary, such as treatment of a contaminant.

To compare the potential clinical desirability of antimicrobial therapy decisions between these RDT platforms, theoretical antimicrobial therapy decisions were made in a blind fashion by two ID-trained clinicians based on the RDT results. They were provided the results of Verigene BC and ePlex BCID panels, baseline clinical information, such as source of infection and multidrug-resistant-organism (MDRO) history, and antibiogram data. To further assist in their decisions, they were also provided a modified UMMS RDT BSI treatment algorithm (Fig. S1).

### Statistical methods.

Positive percent agreement (PPA) between each RDT (either Verigene BC or the ePlex BCID panels) and Vitek MS was determined for on-panel organisms across the tested panels and for each panel. PPA was calculated as 100× the number of true positives divided by the number of true positives combined with false positives. Additionally, two-sided 95% confidence intervals (CIs) were calculated for each PPA (22).

Interobserver agreement between ID clinicians was determined through Cohen’s kappa statistic for both RDT platforms and scores were averaged between decisions. Descriptive statistics included proportions for nominal data and mean (standard deviation [SD]) or median (interquartile range [IQR]) for continuous data. Since DOOR-MAT, used with a partial credit scoring system, is meant to reflect continuous scoring, the results are presented as means and SDs, and comparisons were made using paired *t* tests. Nonparametric analysis would assess the rank of the desirability and negate the continuous scoring aspect of the DOOR-MAT approach (19). All statistical tests were completed using Microsoft Excel (Microsoft Corporation) and SAS version 9.4 (SAS Institute, Cary, NC).

## References

[1] Sullivan KV, Dien Bard J. 2019. New and novel rapid diagnostics that are impacting infection prevention and antimicrobial stewardship. Curr Opin Infect Dis 32:356–364. 10.1097/QCO.0000000000000565. 31135388

[2] Banerjee R, Teng CB, Cunningham SA, Ihde SM, Steckelberg JM, Moriarty JP, Shah ND, Mandrekar JN, Patel R. 2015. Randomized trial of rapid multiplex polymerase chain reaction–based blood culture identification and susceptibility testing. Clin Infect Dis 61:1071–1080. 10.1093/cid/civ447. 26197846 PMC4560903

[3] Avdic E, Wang R, Li DX, Tamma PD, Shulder SE, Carroll KC, Cosgrove SE. 2017. Sustained impact of a rapid microarray-based assay with antimicrobial stewardship interventions on optimizing therapy in patients with Gram-positive bacteraemia. J Antimicrob Chemother 72:3191–3198. 10.1093/jac/dkx267. 28961942

[4] Bookstaver PB, Nimmich EB, Smith TJ, Justo JA, Kohn J, Hammer KL, Troficanto C, Albrecht HA, Al-Hasan MN. 2017. Cumulative effect of an antimicrobial stewardship and rapid diagnostic testing bundle on early streamlining of antimicrobial therapy in Gram-negative bloodstream infections. Antimicrob Agents Chemother 61:e00189-17. 10.1128/AAC.00189-17. 28630187 PMC5571292

[5] Claeys KC, Heil EL, Hitchcock S, Johnson JK, Leekha S. 2020. Management of Gram-negative bloodstream infections in the era of rapid diagnostic testing: impact with and without antibiotic stewardship. Open Forum Infect Dis 7:ofaa427. 10.1093/ofid/ofaa427. 33134414 PMC7585329

[6] Timbrook TT, Morton JB, McConeghy KW, Caffrey AR, Mylonakis E, LaPlante KL. 2017. The effect of molecular rapid diagnostic testing on clinical outcomes in bloodstream infections: a systematic review and meta-analysis. Clin Infect Dis 64:15–23. 10.1093/cid/ciw649. 27678085

[7] Rivard KR, Athans V, Lam SW, Gordon SM, Procop GW, Richter SS, Neuner E. 2017. Impact of antimicrobial stewardship and rapid microarray testing on patients with Gram-negative bacteremia. Eur J Clin Microbiol Infect Dis 36:1879–1887. 10.1007/s10096-017-3008-6. 28534213

[8] Donner LM, Campbell WS, Lyden E, Van Schooneveld TC. 2017. Assessment of rapid-blood-culture-identification result interpretation and antibiotic prescribing practices. J Clin Microbiol 55:1496–1507. 10.1128/JCM.02395-16. 28250000 PMC5405267

[9] Foster RA, Kuper K, Lu ZK, Bookstaver PB, Bland CM, Mahoney MV. 2017. Pharmacists’ familiarity with and institutional utilization of rapid diagnostic technologies for antimicrobial stewardship. Infect Control Hosp Epidemiol 38:863–866. 10.1017/ice.2017.67. 28490386

[10] Ward C, Stocker K, Begum J, Wade P, Ebrahimsa U, Goldenberg SD. 2015. Performance evaluation of the Verigene (Nanosphere) and FilmArray (BioFire) molecular assays for identification of causative organisms in bacterial bloodstream infections. Eur J Clin Microbiol Infect Dis 34:487–496. 10.1007/s10096-014-2252-2. 25311986

[11] Bhatti MM, Boonlayangoor S, Beavis KG, Tesic V. 2014. Evaluation of FilmArray and Verigene systems for rapid identification of positive blood cultures. J Clin Microbiol 52:3433–3436. 10.1128/JCM.01417-14. 25031445 PMC4313169

[12] Arroyo MA, Denys GA. 2017. Parallel evaluation of the MALDI Sepsityper and Verigene BC-GN assays for rapid identification of Gram-negative bacilli from positive blood cultures. J Clin Microbiol 55:2708–2718. 10.1128/JCM.00692-17. 28637912 PMC5648708

[13] Wilson B, Viau R, Perez F, Jiang H, Fowler VG, Chambers HF, Kreiswirth BN, Bonomo RA, Evans SR, ARLG, 2019. Corrigendum to: 1757. Using the desirability of outcome ranking for management of antimicrobial therapy (DOOR-MAT) to assess antibiotic therapy guided by rapid molecular diagnostics (RMD) in bloodstream infection (BSI) caused by Escherichia coli and Klebsiella pneumonia. Open Forum Infect Dis 6:ofz267. 10.1093/ofid/ofz267. 31290855 PMC6608933

[14] Evans SR, Follmann D. 2016. Using outcomes to analyze patients rather than patients to analyze outcomes: a step toward pragmatism in benefit:risk evaluation. Stat Biopharm Res 8:386–393. 10.1080/19466315.2016.1207561. 28435515 PMC5394932

[15] Claeys KC, Schlaffer K, Smith R, Hitchcock S, Jiang Y, Evans S, Johnson JK, Leekha S. 2021. Day at the races: comparing BioFire FilmArray blood culture ID panels to Verigene blood culture in Gram-negative bloodstream infections using DOOR-MAT analysis. Clin Infect Dis10.1093/cid/ciab262. PMC8442774 33772269

[16] Huang T-D, Melnik E, Bogaerts P, Evrard S, Glupczynski Y. 2019. Evaluation of the ePlex blood culture identification panels for detection of pathogens in bloodstream infections. J Clin Microbiol 57:e01597-18. 10.1128/JCM.01597-18. 30487304 PMC6355516

[17] Carroll KC, Reid JL, Thornberg A, Whitfield NN, Trainor D, Lewis S, Wakefield T, Davis TE, Church KG, Samuel L, Mills R, Jim P, Young S, Nolte FS. 2020. Clinical performance of the novel GenMark Dx ePlex blood culture ID Gram-positive panel. J Clin Microbiol 58:e01730-19. 10.1128/JCM.01730-19. 31996444 PMC7098771

[18] Oberhettinger P, Zieger J, Autenrieth I, Marschal M, Peter S. 2020. Evaluation of two rapid molecular test systems to establish an algorithm for fast identification of bacterial pathogens from positive blood cultures. Eur J Clin Microbiol Infect Dis 39:1147–1157. 10.1007/s10096-020-03828-5. 32020397 PMC7225181

[19] Wilson BM, Jiang Y, Jump RLP, Colindres RV, Perez F, Bonomo RA, Evans SR. 2020. Desirability of outcome ranking for the management of antimicrobial therapy (DOOR MAT): a framework for assessing antibiotic selection strategies in the presence of drug resistance. Clin Infect Dis10.1093/cid/ciaa1769. PMC8516503 33245333

[20] Claeys KC, Heil EL, Pogue JM, Lephart PR, Johnson JK. 2018. The Verigene dilemma: gram-negative polymicrobial bloodstream infections and clinical decision making. Diagn Microbiol Infect Dis 91:144–146. 10.1016/j.diagmicrobio.2018.01.012. 29449043

[21] Evans SR, Rubin D, Follmann D, Pennello G, Huskins WC, Powers JH, Schoenfeld D, Chuang-Stein C, Cosgrove SE, Fowler VG, Lautenbach E, Chambers HF. 2015. Desirability of outcome ranking (DOOR) and response adjusted for duration of antibiotic risk (RADAR). Clin Infect Dis 61:800–806. 10.1093/cid/civ495. 26113652 PMC4542892

[22] Food and Drug Administration. 2007. Statistical guidance on reporting results from studies evaluating diagnostic tests. FDA, Silver Spring, MD.

