# Supplementary material for: Comparing the Clinical Utility of Rapid Diagnostics for Treatment of Bloodstream Infections Using Desirability of Outcome Ranking Approach for the Management of Antibiotic Therapy (DOOR-MAT)
Source: Antimicrob Agents Chemother. 2021 Aug 17;65(9):e00441-21. doi: 10.1128/AAC.00441-21 (PMC8370220; doi:10.1128/AAC.00441-21)
Supplement: Supplemental file 1 — Supplemental Figure S1 and Table S1. Download AAC.00441-21-s0001.pdf, PDF file, 0.4 MB [file aac.00441-21-s0001.pdf]

Part 1: Gram-positive BSI

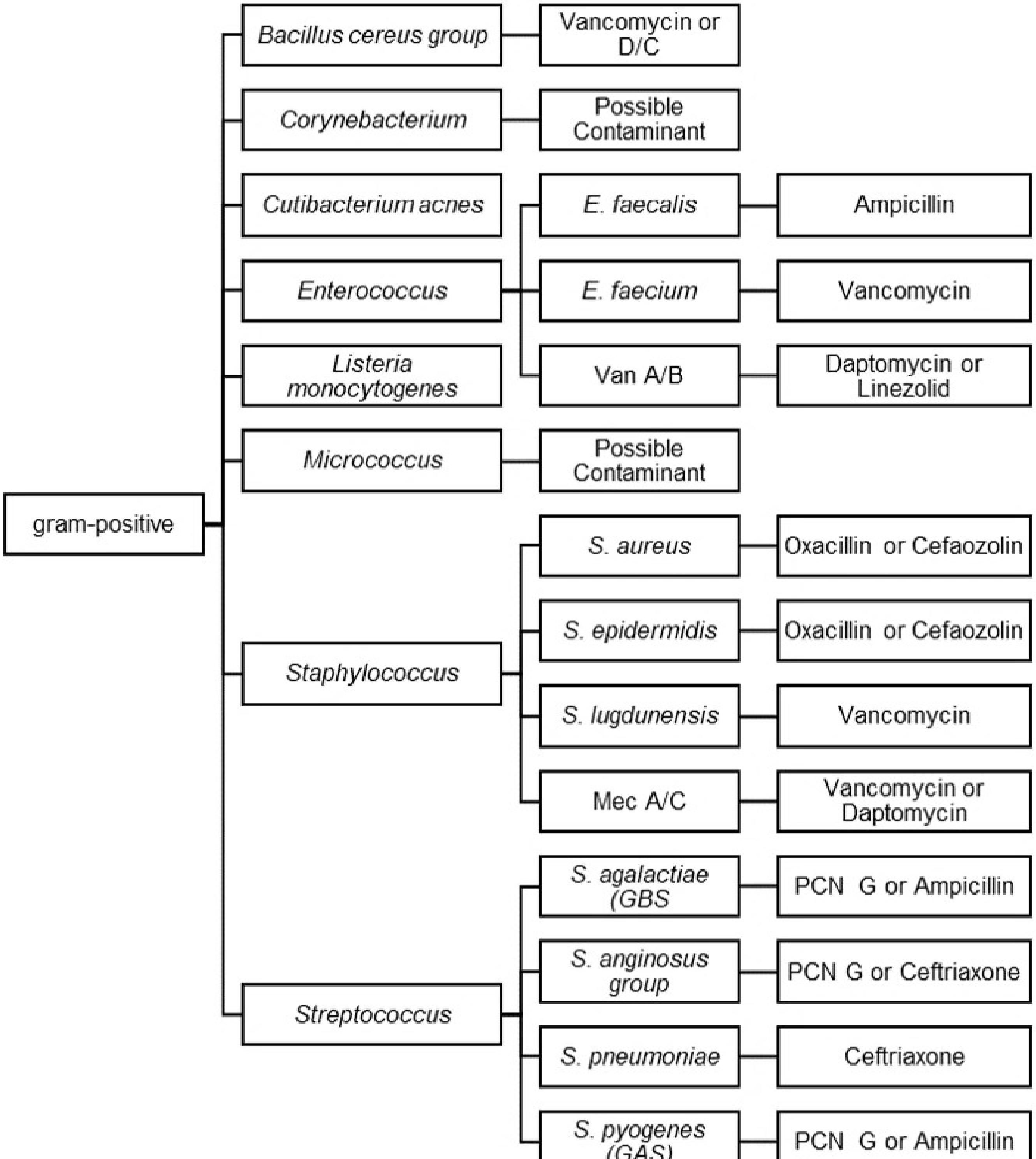

Supplementary Figure: Modified University of Maryland Medical System RDT Treatment Algorithm for BSI

Part 2: Gram-negative BSI

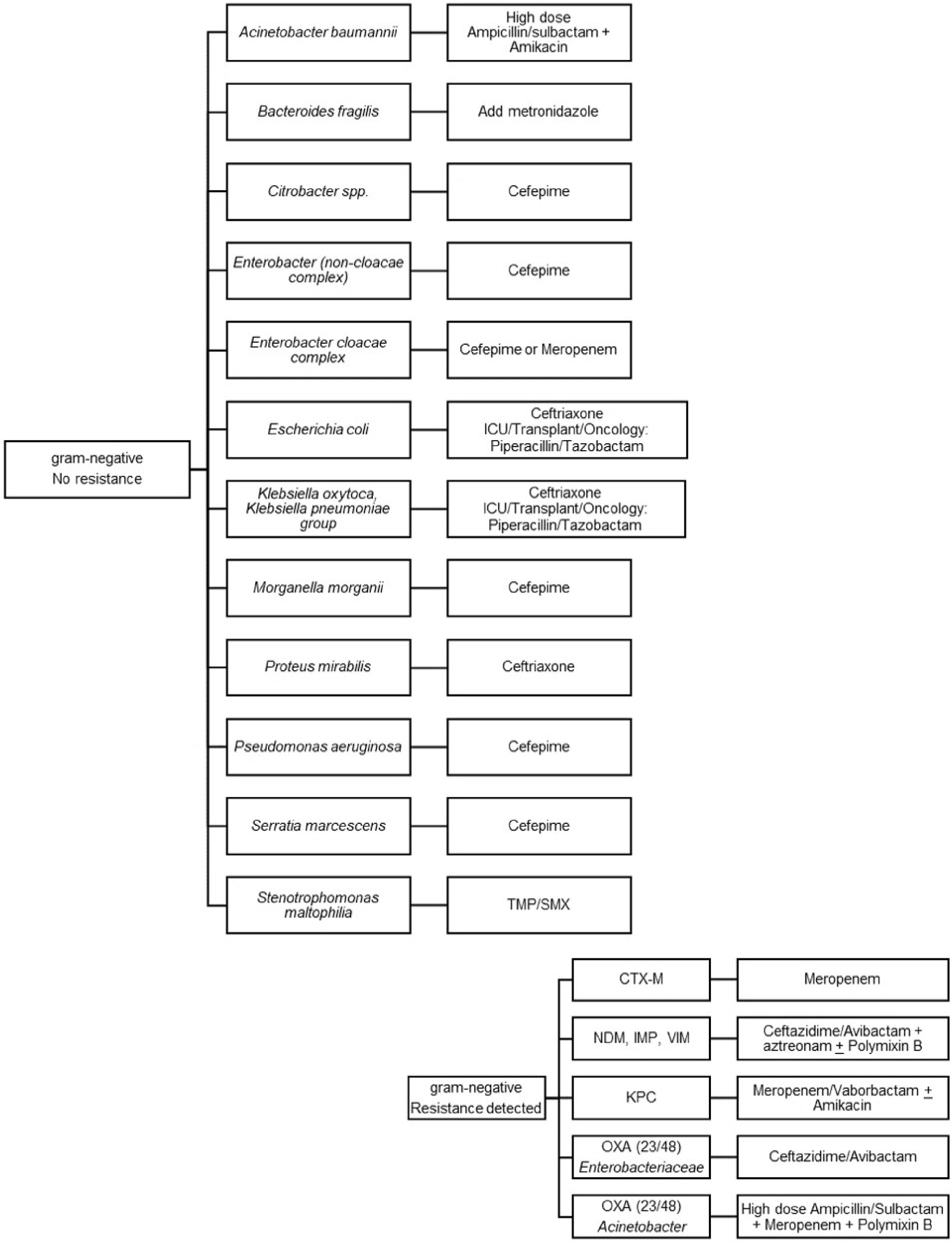

Recommendations based on local resistance patterns, formulary availability, and available clinical evidence

Supplementary Table 1: Summary of Discrepancy Testing Results

Gram-stain	ePlex BCID Panels	VITEK 2/MS	Discrepancy	Testing	Resolution
GPCCl	*Staphylococcus*<br>*S. epidermidis*	*S. aureus*<br>Methicillin S	Unexpected *S. epidermidis* on ePlex; *S. aureus* not detected	Repeat ePlex BCID-GP rom blood & isolated colonies<br>16S Sequencing	*S. epidermidis*<br>*S. epidermidis* true positive on ePlex
GPCCl	*Enterococcus*<br>Pan Gram-Negative	*E. gallinarum*<br>Vancomycin R	Unexpected Pan Gram Negative positive, *vanA/vanB* not detected by ePlex BCID-GP	Repeat ePlex BCID-GP from blood & isolated colonies<br>ePlex BCID-GN from blood	*vanA/vanB* true negative on ePlex<br>Pan Gram-Negative false positive on ePlex
GNR	*Enterobacter cloacae* complex	*Enterobacter cloacae*<br>*S. maltophilia*	*S. maltophilia* not detected on ePlex BCID-GN	Repeat ePlex BCID-GN from blood & isolated colonies	*S. maltophilia* true negative on ePlex
GNR	*E. coli*<br>Pan Gram-Positive	*E. coli*	Unexpected Pan Gram-Positive on ePlex BCID-GN	Repeat ePlex BCID-GN from blood & isolated colonies<br>ePlex BCID-GP from blood	Pan Gram-Positive false positive on ePlex
GNR	Not Detected	*Acinetobacter junii*<br>*S. maltophilia*	*S. maltophilia* not detected on ePlex BCID-GN	Repeat ePlex BCID-GN from blood & isolated colonies<br>16S Sequencing	*Acinetobacter junii*<br>*Stenotrophomonas acidaminiphila*<br>*S. maltophilia* true negative on ePlex
GNR	*P. aeruginosa*<br>*S. maltophilia*	*P. aeruginosa*	Unexpected *S. maltophilia* detected on ePlex BCID-GN	Repeat ePlex BCID-GN from blood & isolated colonies<br>16S Sequencing	*P. aeruginosa*<br>*S. maltophilia* false positive on ePlex
GPCPr	*Enterococcus*<br>*E. faecalis*<br>*vanA*	*E. faecium*<br>Vancomycin S	Unexpected *vanA* on ePlex BCID-GP	Repeat ePlex BCID-GP from blood & isolated colonies	*vanA* false positive on ePlex
GNR/GPR	*K. pneumoniae*<br>Pan Gram-Positive	*K. pneumoniae*	Needs to be tested on ePlex BCID-GP	Repeat ePlex BCID-GN from blood & isolated colonies<br>ePlex BCID-GP from isolated colonies	*Enterococcus* and *E. faecalis* detected<br>Pan Gram-Positive true positive on ePlex
GNR	Pan Gram-Positive	*E. coli*	*E. coli* not detected by ePlex BCID-GN<br>Needs to be tested on ePlex BCID-GP	Repeat ePlex BCID-GN from blood & isolated colonies<br>ePlex BCID-GP from blood & isolated colonies	*E. coli* false negative on ePlex<br>Pan Gram-Positive false positive on ePlex
GNR	Pan Gram-Positive	*P. aeruginosa*<br>CoNS	*P. aeruginosa* not detected on ePlex BCID-GN<br>Needs to be tested on BCID-GP	Repeat ePlex BCID-GN from blood & isolated colonies<br>ePlex BCID-GP from blood & isolated colonies<br>16S Sequencing	*P. mendocina*<br>*P. aeruginosa* true negative on ePlex<br>*S. epidermidis* detected
GNR	*K. pneumoniae*<br>Pan Gram-Positive	*K. pneumoniae*	Needs to be tested on ePlex BCID-GP	ePlex BCID-GP from blood & isolated colonies	*Enterococcus* and *E. faecalis* detected<br>Pan Gram-Positive true positive on ePlex

CoNS = coagulase negative staphyloccous; GPCCl = gram positive cocci in clusters; GPR = gram-positive rod; GNR = gram-negative rod; GPCPr = gram-positive cocci in pairs; R = resistant; S = susceptible;
